# The motivational cost of inequality: Opportunity gaps reduce the willingness to work

**DOI:** 10.1371/journal.pone.0237914

**Published:** 2020-09-04

**Authors:** Filip Gesiarz, Jan-Emmanuel De Neve, Tali Sharot

**Affiliations:** 1 Affective Brain Lab, Department of Experimental Psychology, University College London, London, United Kingdom; 2 Saïd Business School, University of Oxford, Oxford, United Kingdom; FAME|GRAPE, POLAND

## Abstract

Factors beyond a person’s control, such as demographic characteristics at birth, often influence the availability of rewards an individual can expect for their efforts. We know surprisingly little how such differences in opportunities impact human motivation. To test this, we designed a study in which we arbitrarily varied the reward offered to each participant in a group for performing the same task. Participants then had to decide whether or not they were willing to exert effort to receive their reward. Across three experiments, we found that the unequal distribution of offers reduced participants’ motivation to pursue rewards even when their relative position in the distribution was high, and despite the decision being of no benefit to others and reducing the reward for oneself. Participants’ feelings partially mediated this relationship. In particular, a large disparity in rewards was associated with greater unhappiness, which was associated with lower willingness to work–even when controlling for absolute reward and its relative value, both of which also affected decisions to work. A model that incorporated a person’s relative position and unfairness of rewards in the group fit better to the data than other popular models describing the effects of inequality. Our findings suggest opportunity-gaps can trigger psychological dynamics that hurt productivity and well-being of all involved.

## Introduction

Randomness plays a surprisingly important role in determining the barriers and opportunities encountered by individuals on their path to a prosperous life [1]. Country of birth alone explains 66% of global variation in living standards [2]. Other non-meritocratic factors, such as zip code [3], parental socio-economic status [4], gender [5], or a person’s name [6] have been shown to have a significant effect on earnings, even after accounting for inter-individual differences in merit. Economic inequality arising due to random circumstances is often viewed as unfair [7], and previous studies have shown that people support redistribution of wealth in such situations [8]. However, much less is known about how opportunity gaps influence human motivation. Such knowledge could shed light on psychological mechanisms that lead to differences in aspirations, that in turn might contribute to higher unemployment [9–11] and lower university application rates of people from disadvantaged backgrounds [12–14]. Here, we examine how randomly assigned unequal reward prospects can influence a person’s willingness to exert effort in exchange for rewards–a proxy measure of motivation in labour supply decisions.

Due to a lack of experimental research on the impact of inequality on motivation, the underlying mechanisms of this relationship remain unknown. We hypothesize that arbitrary differences in opportunities to earn rewards can negatively impact not only disadvantaged individuals but also those who are offered relatively high rewards. This is because facing opportunity gaps can involve two separate mechanisms: relative comparisons and reactions to unfairness, representing self-regarding and group-regarding reactions to inequality, respectively [15]. First, because people engage in spontaneous social comparisons, evaluating their rewards relative to those of others [16–19], opportunity gaps can increase motivation to pursue rewards of those offered relatively high rewards and reduce the motivation of those offered relatively low rewards. However, at the same time people may have a negative response to the unfairness of arbitrary distributions of rewards in their group regardless of which side of the distribution they are at, and be less willing to pursue rewards in situations that are unfair. Indeed, it has been shown that subjects are less happy when they themselves win in a gambling task, but the other subject loses, in comparison to when both subjects win [20]. We hypothesize that such a negative reaction may have consequences beyond a person’s affective state. Specifically, negative feelings can lead to apathy as well as a reduction in the subjective value of rewards [21], leading to a reduced motivation of all members of the group. Thus, individuals at the bottom of the distribution may be negatively affected twice, first due to their lower relative position and second due to their reaction to unfair distribution.

We formalize the above hypotheses in a model that characterizes the motivational response to rewards as a linear combination of reward’s absolute value, relative value, and statistical dispersion of all rewards in the group. Based on the law of decreasing marginal utility, we assume that absolute reward has a non-linear effect on decisions to engage in an effort to earn the reward [22]. As previous studies have shown that people have a tendency to engage in ordinal rather than absolute comparisons [23], we define the relative value of rewards as the rank of the offered reward. Statistical dispersion is calculated in our model as Gini coefficient, following other studies suggesting a relation between this measure and well-being in national surveys [24].

In three experiments, we were able to dissociate and quantify the influence unfairness, reward’s rank, its absolute value, while studying them independently from other factors that are often associated with opportunity gaps, such as demographics or stereotypes. In all three studies, participants made decisions on whether to exert cognitive effort in exchange for a reward while observing the rewards offered to others for completing the same task. In these experiments we manipulated: (i) the deviation of payments in the group from an equal distribution (thereafter ‘unfairness’), and (ii) the relative position of the offer in the distribution (thereafter ‘rank’). Experiment 1 aimed to establish if the motivation to work for rewards is influenced by unfairness and rank of offered rewards. Experiments 2 and 3 aimed to test the mechanisms underlying the influence of relative value and unfairness on motivation, including the mediating role of emotions, and the moderating role of uncertainty.

## Methods Experiment 1

### Overview

**Fig 1:**
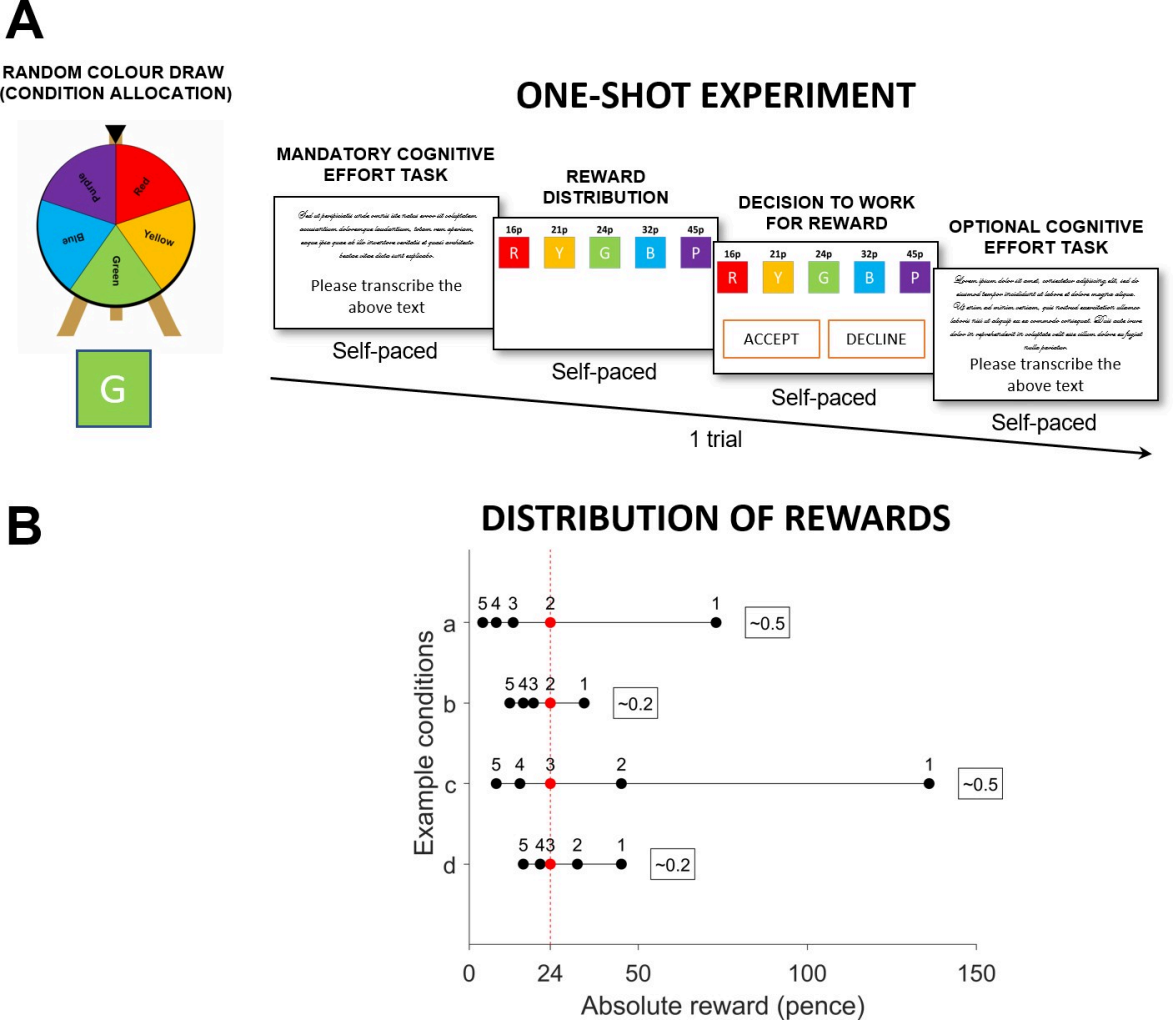
Behavioural task in Experiment 1. (A) The online task had a one-shot design. The task was advertised on an online labour market platform as a simple transcribing job. After completing the mandatory cognitive task, participants were informed that they would have an opportunity to complete an optional transcribing task for a randomly drawn fee. The random draw was determined by spinning a wheel of fortune that assigned a participant one out of five different colours. After the colour assignment, participants were presented with the reward offers for all five colours, and were told that the other rewards were assigned to other people who drew those colours. Participants then decided to either accept or decline the reward offer for the optional task. If they accepted it, they had to transcribe an additional text. If they declined it, the task ended, and they were granted their base fee. Unbeknownst to participants, the reward offer was always equal to £0.24, and the random colour assignment determined if the offer was presented either as 5^th^, 4^th^, 3^rd^, 2^nd^ or 1^st^ best reward. Independently, participants were randomly assigned to one of two levels of unfairness (Gini coefficient = 0.3 or Gini coefficient = 0.5) of reward distribution. (B) The task had a 5x2 design (10 conditions in total): two levels of inequality, and five levels of relative value. Each participant viewed only one of these conditions. Panel B illustrates example conditions. The example a) shows a situation where £0.24 was presented as the second-best reward in an unfair context, and example b) where it was presented as the second-best reward in a fair context. The example c) shows a situation where £0.24 was presented as the middle reward in an unfair context, and the example d) shows a situation where £0.24 was presented as the middle reward in a fair context.

Experiment 1 used a one-shot design (Fig 1) and was conducted on Prolific—an online labour market platform. Participants were offered £0.24 for an optional task of transcribing 1/3 of a page of text from a displayed image and were made to believe that this reward offer was drawn at random. The offered reward was displayed in the context of 4 other rewards assigned randomly to other workers on the platform. Seven hundred participants were assigned to separate conditions in a 2x5 design that determined the context of their offered reward: the five rewards could be either relatively equally distributed or unequally distributed, and participant’s reward of £0.24 could be presented either as 5^th^, 4^th^, 3^rd^, 2^nd^ or 1^st^ best-offered reward.

### Participants

In experiment 1, seven hundred participants were recruited to take part in the online study, spread evenly across ten conditions (70 participants per condition). All participants provided written informed consent. The experiment was approved by the UCL ethics committee. Participants were recruited through the Prolific platform–an online platform for offering web-based tasks. Eighty participants were excluded due to failing attention check that asked them about the colour that they have been assigned to. This exclusion criterion was necessary, as the colour indicated which reward a participant was offered. All participants in the online task were currently UK residents (mean age 26.2[5.0], age range 18–35, 487 women). The average self-identified political orientation was 4.61(1.61) on a scale ranging from 1 (extremely right-wing) to 7 (extremely left-wing), significantly more left-wing than the centre of the scale (t(699) = 18.27, p < 0.001).

### Procedure

Participants responded to an ad on Prolific platform that recruited people for a short transcribing task–a common task on online labour markets. The display of the advertisement was restricted to current UK residents aged 18–35. After signing up to complete the task, participants were informed that the task will consist of a mandatory transcribing task, for which they will be paid the advertised wage (£0.25), and an optional transcribing task for which they will be paid a bonus payment. The mandatory transcribing task required participants to transcribe a 1/5 of a page from an old cookbook. The optional task was to transcribe a different text from the same cookbook, which was approximately 3 times longer. The instructions emphasized that participants had to be 99% accurate to receive the bonus payment. There was no time limit.

They were also informed that the wage for the optional task would be randomly drawn. The random draw was determined by a wheel of fortune that after spinning for 3 seconds picked one colour out of 5 colours. After the participant was assigned one of 5 colours, the bonus wages for the optional task were revealed all at once for all 5 colours. Participants were told that information about the other wages was displayed to inform other Prolific users who drew different colours. Unbeknownst to participants, the offered wage for the optional task was always equal to £0.24, and each participant was assigned to one condition in a 2x5 design that determined the context in which the reward was displayed. In particular, the reward could be presented either as 5^th^, 4^th^, 3^rd^, 2^nd^ or 1^st^ best reward, and was presented either in a context of a roughly fair distribution of rewards between participants (corresponding to a 0.2 Gini coefficient) or an unfair distribution (corresponding to a 0.5 Gini coefficient). Full list of reward distributions is included in the S2 Table. After seeing the reward offers, participants had to decide to either accept or reject the optional task. If they decided to accept it, they had to transcribe an additional text and were paid their bonus wage (£0.24) plus base wage (£0.25). If they decided to reject it, they were paid just their base wage (£0.25).

### Data analysis

To test the influence of reward’s rank and unfairness of the distribution, we used a Generalized Linear Model (GLM) that included decisions to work as the categorical dependent variable, and unfairness (measured as Gini coefficient) and rank (normalized to range from 0 to 1, for lowest and highest rank respectively) as independent variables. Both independent variables were standardized prior to the analysis. The GLME model assumed a binomial distribution of the dependent variable.

Participant’s offer rank was normalized to range from 0 to 1 as follows:

$${Rank}_{t}=\frac{i-1}{n-1}$$

Where *i* is the reward offer index in a set of offers ordered from lowest to highest and *n* is the number of participants in the group (in our case 5). The above rank measure assigns 1 to the person with the best offer, 0 to the person with the lowest offer, and 0.5 to the person with the intermediate offer. Unfairness was measured as the Gini coefficient, calculated as follows:

$$Unfairness=\frac{\frac{1}{n^{2}}\sum_{i=1}^{n}\sum_{j=1}^{n}\left|x_{i}-x_{j}\right|}{2\bar{x}}$$

Where *n* is the number of participants in the group, *x*_*i*_ and *x*_*j*_ is the reward offers received by each person, and $\bar{x}$ is the mean reward offer.

**Fig 2:**
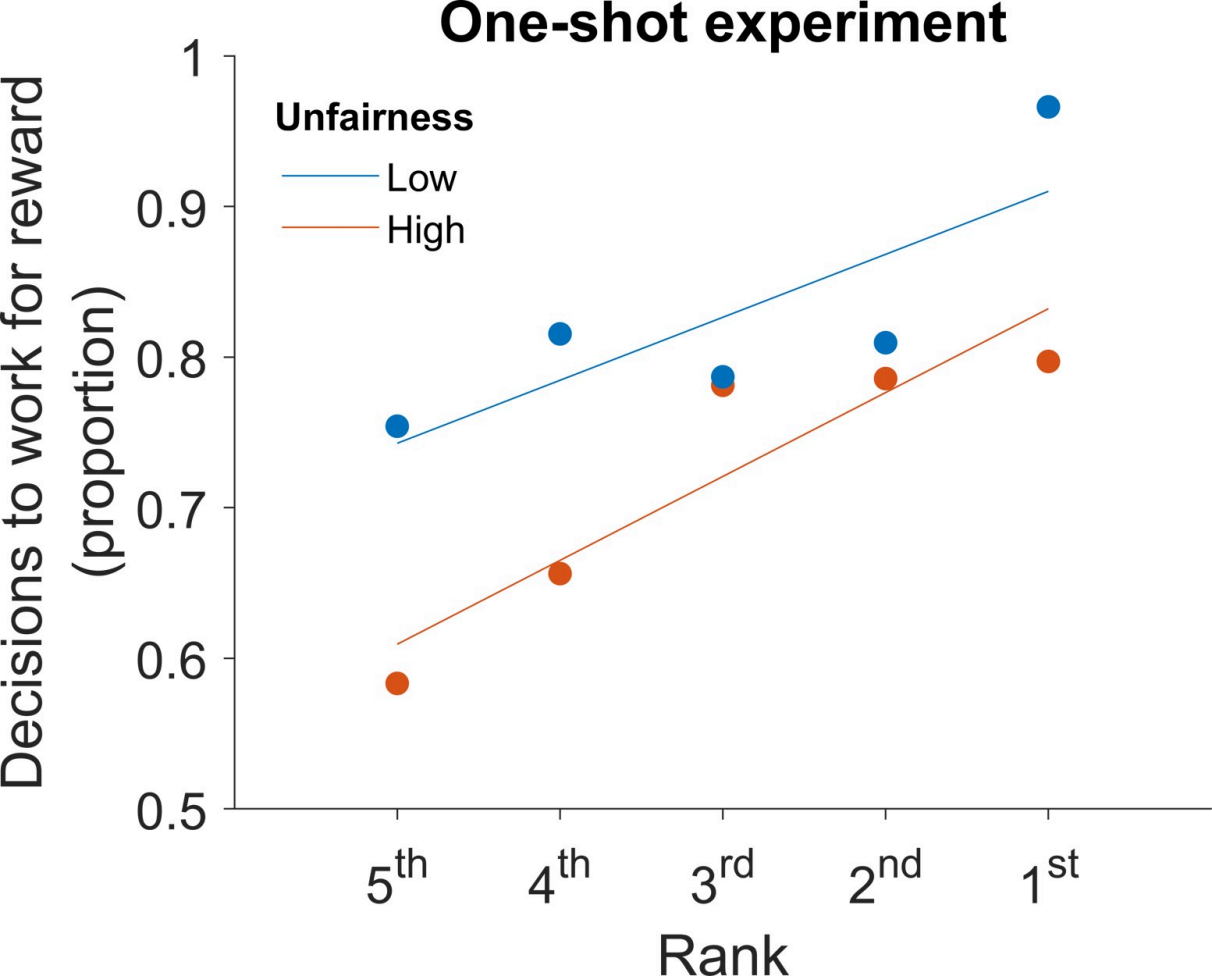
Motivation to work is higher when the distribution of rewards is fair and the rank of the reward is high, despite the same level of absolute reward. The plot illustrates the results from a one-shot experiment conducted on an online labour market platform. Each dot represents the proportion of participants who decided to perform an additional task for a bonus reward of £0.24, which was presented either in a relatively fair (blue) or unfair (red) context, and either as the 5^th^, 4^th^, 3^rd^, 2^nd^ or 1^st^ best reward. The lines represent the best fitting line based on the Ordinary Least Squares method. Participants were more likely to accept the offer of £0.24 when its rank was high than when it was low, and when the rewards of all participants were fairly distributed than when they were unfairly distributed.

To illustrate the results from experiment 1, we plotted the number of participants who decided to pursue additional reward divided by the number of all participants in the condition separately for each rank and level of unfairness (Fig 2).

## Results Experiment 1

Overall, 77.33% of participants decided to perform the optional task in exchange for an additional fee of £0.24. However, we found that participants were less willing to work for the additional reward when they believed that the distributions of offered rewards were unfair vs. fair (β = -0.31, p < 0.01), and when the rank of their reward was low vs. high (β = 0.40, p < 0.001), despite absolute reward being the same across all conditions in this experiment (Fig 2). On average, an increase of 0.3 in Gini coefficient resulted in 10.6% less accepted offers, and increase of one rank resulted in 5.3% more accepted offers.

### Procedure for onsite experiments (Experiments 2 & 3)

#### Overview

**Fig 3:**
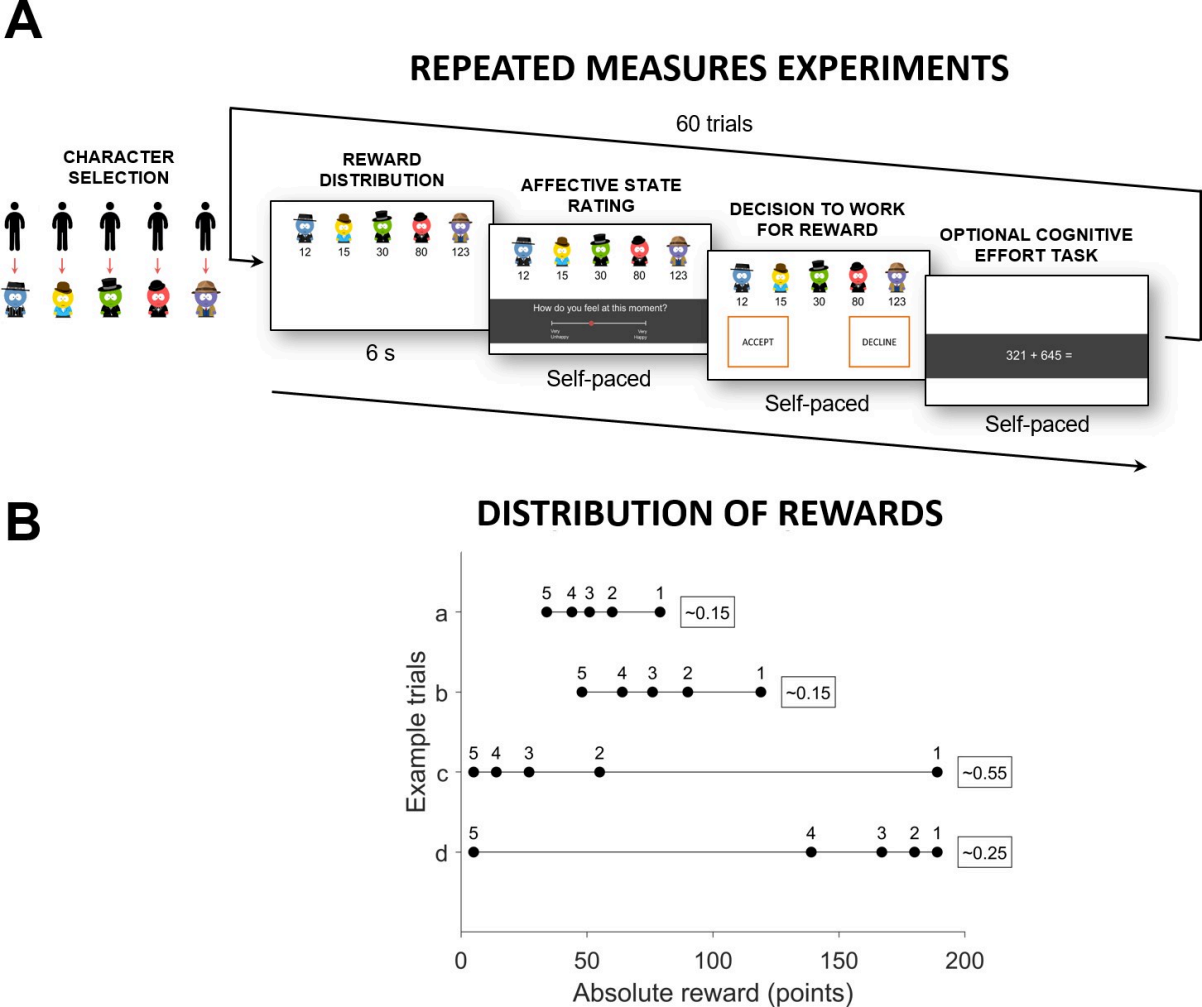
Behavioural task in Experiment 2 and 3. (A) Both Experiment 2 and 3 used a repeated measures design. Participants were invited to the lab in groups of five. To easily identify themselves during the experiment, each participant selected a cartoon avatar that would represent them throughout the task. They then retired to individual cubicles to complete the study. There were 60 trials in total. Each trial started with a display of all participants’ reward offers, that differed in rank, absolute reward, and level of unfairness between participants. After seeing the distribution of rewards, participants rated their current feelings and indicated whether they were willing to exert cognitive effort for their offered reward on that specific trial. If they decided to do so, they would complete three mathematical problems. If not, they would move on to the next trial. If a participant gave an incorrect answer to the mathematical problem, they would have to solve an additional one, until they completed three problems correctly. (B) For the repeated measure experiments, we created 30 income distributions based on a log-normal probability density function (corresponding to 10 levels of Gini index uniformly distributed between 15 and 55, with 3 different median values) Log-normal distribution approximates reward distributions encountered in real-world, such as income distributions within countries [25] and companies [26]. Because these distributions are always positively skewed, we also created 30 distributions that were negatively skewed and a mirror image of the positively skewed distributions. For illustration purposes, we plot four of these distributions. Each dot on the line represents one of five reward offers presented to participants. Numbers above the dots refer to the reward’s rank. Numbers in the rectangles refer to unfairness level, expressed in the Gini coefficient. Distribution a) is an example of a fair positively skewed distribution with a low median reward; distribution b) is an example of similarly fair distribution, but with a higher median value; Distribution c) is an example of an unfair positively skewed distribution, and distribution d) is an example of a negatively skewed distribution that is a mirror image of c).

Experiments 2 and 3 followed a similar logic as experiment 1, but used a repeated measure design (Fig 3), in which the same person was exposed to different distributions of rewards. Repeated measure designs achieve greater statistical power with fewer participants, allowing us to test more efficiently a larger number of hypotheses regarding the mechanisms underlying the effects observed in Experiment 1. Experiments 2 and 3 aimed to test the robustness of the effects observed in Experiment 1 when translated to a different context. Both experiments included a greater variety of distributions (both positively skewed and negatively skewed) and a different cognitive effort task. Different distributions allowed us to test the predictions of different models describing the impact of inequality on the evaluation of rewards. Additionally, the experiments: a) gathered information about participants’ current feelings after seeing the distribution of rewards, allowing us to test if the observed effects are mediated by the impact of rank and unfairness on person’s emotional state, and b) manipulated the uncertainty about the value of rewards, by either introducing a known (Experiment 2) or an unknown (Experiment 3) exchange rate of earned points with £, allowing us to test if reliance on the social context in one’s decisions to work is moderated by uncertainty.

#### Participants

In Experiment 2 and 3, one hundred and ten participants from University College London subject pool were recruited to take part in two onsite studies: sixty in experiment 2 (mean age 22.1[3.2], age range 18–35; 38 women) and fifty in experiment 3 (mean age 21.4[2.0]; age range 18–35; 34 women). All participants provided written informed consent. The experiment was approved by the UCL ethics committee. Across these two experiments, 67% of participants originated from Western countries. The average self-identified political orientation was 3.52(1.38) on a scale ranging from 1 (extremely right-wing) to 7 (extremely left-wing) and was not significantly different from the centre of the scale (t(87) = 0.12, p = 0.91). All participants started with an initial endowment of £10 and were paid an additional bonus based on their decision to accept or reject reward offers in exchange for performing a cognitive task in one randomly selected trial. Participants who accepted all reward offers were excluded from the data analysis as we could not identify the factors influencing their decisions due to lack of behavioral variability, beyond the fact that they were maximizing their bonus reward at the end (eight subjects in experiment 2 and seven subjects in experiment 3), leaving 52 and 43 participants in each experimental sample respectively. None of the subjects rejected all offers.

#### Procedure

In both experiments, we invited participants to the lab in groups of five (N = 110 in total). To easily identify themselves during the task, participants were asked to choose a cartoon avatar that would represent them in the study. A randomly drawn lot number determined the order of choosing avatars. Participants were informed that each person will be offered a different reward on each trial and that these rewards were randomly decided on each trial by a computer program. Next, participants retired to separate cubicles where they were given additional instructions.

Participants first completed one practice trial. Both experiments consisted of 60 trials. In each of 60 trials, we presented to participants the reward points offered to each of the five members of the group on that trial. On each trial, we independently manipulated: (i) the deviation of payments in the group from an equal distribution (‘*unfairness’*), (ii) the rank of the reward offered to each person within the group (ranging from 1 to 5 - ‘*rank’*) and (iii) the absolute reward offered (i.e., points—‘*absolute reward’*).

We created 60 different distributions of reward offers in total and presented them in random order. We generated 30 reward distributions based on a log-normal probability density function. Log-normal distribution was chosen as it fits closely real-world income structures within firms [26] and countries [25]. To vary the levels of reward magnitude range and statistical dispersion we used a combination of 3 different median values (0.55, 1, 1.45) and ten different standard deviations, corresponding to values of the Gini coefficient varying uniformly from 20 to 65, resulting in 30 different distributions. Log-normal distributions are always positively skewed. To generalize our findings, we also included 30 negatively skewed distributions that were a mirror-image of the positively skewed distributions by applying the following transformation of representative values:

$$x_{positive}=\{x_{1},x_{2},x_{3},x_{4},x_{5}\}$$

$$x_{negative}=|x_{positive}-max(x_{positive})|+\min\left(x_{positive}\right)$$

Where *x*_*n*_ is subject *n* payment offer in each trial, *x*_*positive*_ and *x*_*negative*_ are payment offers of all participants in trials with positively and negatively skewed distributions, respectively.

To generate reward offers representative of the above distributions, we used an inverse cumulative density function of these distributions, which assigns maximal pay value earned by each percent of the population. We next took an average pay from subsequent 20 percentiles of this function, with the exclusion of top 1 percentile, resulting in 5 values reflecting an average pay of each 20% of the population. The last percentile was excluded as it approaches infinity. Unfairness was quantified based on these 5 representative values. To introduce variability to the middle pay (that otherwise would be the same for all distributions generated from the same median value) we additionally subtracted a number between 0 and 9 from each representative value in each distribution (in each distribution the same number was subtracted for each value). This resulted in the pay offers shown in S1 Table.

After seeing the distribution of reward offers, participants then rated their feelings by clicking on a continuous sliding scale ranging from very unhappy to very happy. The slider started in the middle of the scale on every trial. After the feeling ratings, participants indicated whether they were willing to complete three mathematical problems to earn their reward. If they decided to do so, they were asked to solve the problems (the instructions emphasized that the mathematical problems were the same for all). If they decided not to, they would move on to the next trial. Each problem required adding two 3-digit numbers. To ensure equal difficulty of mathematical problems throughout the task, each addition had exactly two carryovers (sum of ones, tens or hundreds greater than 10). E.g., problems included sums like 118 + 197. If participants provided an incorrect answer, they had to solve an additional problem. Participants continued until they got three problems correct. On average, 89% of attempts were correct, and it took subjects 17 seconds (SD = 7.56s) on average to solve each problem.

At the end of the study, we selected one trial at random for compensation–a common procedure used to avoid the effects of reward accumulation during the task [27]. If the participant had decided not to work on that trial, no bonus reward was received. If the participant decided to work for a reward on that trial, they would receive the reward offered on that trial. The decision of whether to work did not influence the rewards offered on future trials or pay-out of other members of the group. This information was emphasized in the instructions, and participants had to pass a comprehension check to ensure that they understood the details of the task.

The difference between the two onsite experiments was that in one experiment the participants knew the exchange rate between reward points offered and Great British Pounds (1 point was worth £0.04), in the other experiment it was unknown and said to differ on each trial (ranging from £0.001 to £0.08). The total bonus reward (after exchanging earned points from a selected trial to £) could range from £0 to £18.64 in Experiment 2 and from £0 to £37.28 in Experiment 3. We hypothesized that when the value of points was unknown, participants would rely more heavily on social context when deciding to work for a displayed reward. We replicated the core findings across both studies. Thus, we initially report results from the combined dataset, and then formally test if the effects differed in strength between both experiments. A separate analysis of each dataset is presented in the Supporting Information.

### Data analysis

Although participants on average accepted 54% of reward offers in experiment 2 and 3, we found a considerable variability between participants, with some participants accepting/rejecting as little as just one offer, limiting inferences that can be drawn from a single participant. To account for this issue, as well as within-subject correlations of responses related to repeated measures in our design, we used Generalized Linear Mixed Effects (GLME) model approach, in which fixed effects describe the effect common for all participants and random effects describe idiosyncrasies specific for an individual. The GLME model included decisions to work as the categorical dependent variable and assumed a binomial distribution of the dependent variable. The independent variables were unfairness (measured as Gini coefficient), rank (normalized to range from 0 to 1, for lowest and highest rank respectively), and reward magnitude (expressed as a power function, see below). All variables were standardized prior to the analysis. Following methodological recommendations by Barr and colleagues [28], all models included fixed and random effects for intercept and all independent variables.

Rank and unfairness were calculated as in Experiment 1. To account for a possibility of diminishing marginal utility of each additional awarded point, we tested if the effect of reward magnitude was better expressed as a linear or a power function (as it is in the prospect theory [22]):

$$Reward\;magnitude\;utility={x_{i}}^{\rho }$$

Where *x* is the reward offer, and *ρ* represents parameter describing the curvature of the reward function, ranging from 0 to 1 (at which point it is linear). To fit the above function, we estimated non-linear mixed-effects model with stochastic Expectation-Maximization algorithm [29]. The *ρ* value maximizing the *R*^*2*^ of the model describing the relationship between reward magnitude and motivation to work (including the variables listed in the section below) was equal to 0.43, suggesting a non-linear relationship between absolute reward and its value, and was subsequently used in all analyses.

We additionally tested if skewness of the distribution could separately influence participants’ decisions, by including in the above model an Adjusted Pearson’s Coefficient of Skewness, calculated as follows:

$$Adjusted\;Pearson^{\prime }s\;Coefficient\;of\;Skewness=\frac{\sqrt{n(n-1)}\frac{1}{n}\sum_{i=1}^{n}{\left(x_{i}-\bar{x}\right)}^{3}}{(n-2){\left(\sqrt{\frac{1}{n}\sum_{i=1}^{n}{\left(x_{i}-\bar{x}\right)}^{2}}\right)}^{3}}$$

Where $\bar{x}$ is the average reward offer, *n* is the number of participants in the group, *x*_*i*_ is the reward offer received by each person.

**Fig 4:**
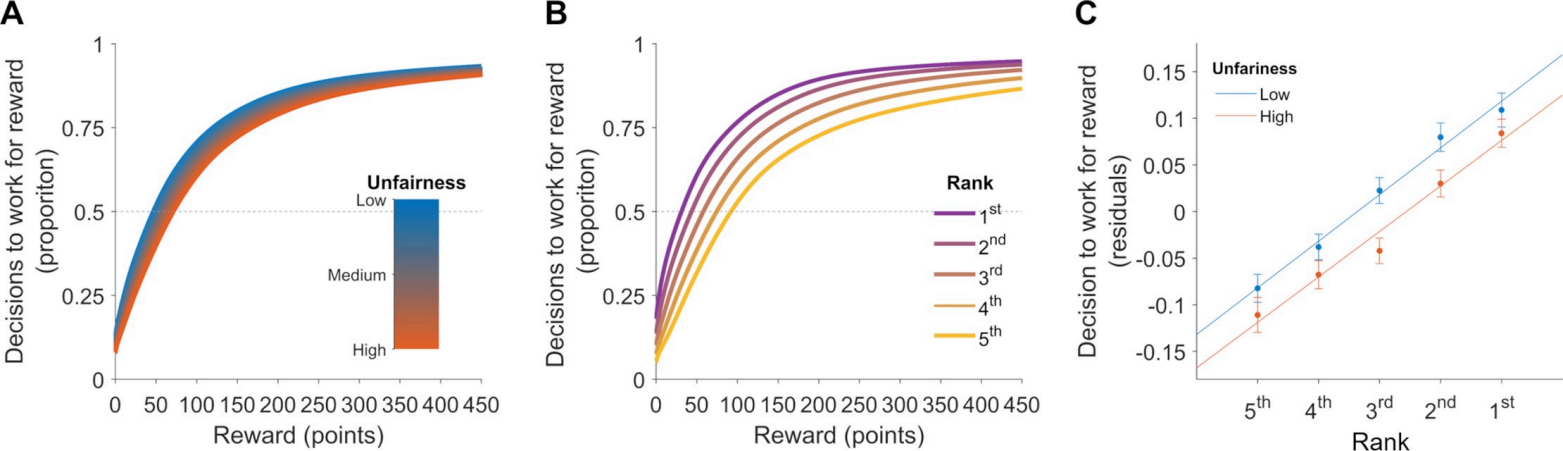
Motivation to work is higher when the distribution of rewards is fair; the rank is high, and the absolute reward is high. To illustrate the effect of factors influencing the motivation to work in repeated measures experiments, we plotted the probability of participants’ decision to work from a GLME model predicting choice from reward magnitude and either different levels of (A) unfairness or (B) rank. (C) We also plotted average residuals for the five rank categories and two levels of unfairness from a GLME model predicting choice just from absolute reward and trial number. We observe that participants are more likely to decide to work **when (A, C) rewards** are fairly distributed and (B, C) when the rank is high than low. Error bars = SEM.

To illustrate the size of the effect of unfairness and rank we plotted predicted values of the above GLME model across different levels of unfairness (Fig 4A) and separately, across different ranks (Fig 4B), with the effect of trial number, rank (only for Fig 4A) and unfairness (only for Fig 4B) set to 0. To illustrate the effect of unfairness and rank in isolation from reward magnitude (Fig 4C), we estimated the probability of pursuing rewards on each trial from a GLME model including absolute reward and trial number (with other factors fixed to 0). We then calculated the residuals, by subtracting observed decisions and their predicted probability. We categorized residuals into 5 ranks and two levels of unfairness (based on the middle value of the tested range) and calculated the average residual value for each participant within each category and plotted the averages over participants within each category.

The fit of the model including rank and unfairness was compared to two other popular models describing the effects of inequality on the evaluation of reward: the adaptation model [30], and inequality aversion model [31]. All compared models included absolute value as one of the independent variables. Adaptation model additionally included the difference between absolute value of the offered reward and the average reward offered to all people in the group on a specific trial. Inequality aversion additionally model included advantageous and disadvantageous inequality, calculated as follows [31]:

$$Advantaegous\;inequality=\sum_{j=1}^{n}max|x_{i}-x_{j},0|$$

$$Disadvantageous\;inequality=\sum_{j=1}^{n}max|x_{j}-x_{i},0|$$

Where *x*_*i*_ is an individual’s payment offer and *x*_*j*_ are payment offers received by other group members. All models were compared based on their Bayesian Information Criterion (BIC) which simultaneously assesses the model’s fit, while penalizing it for its complexity.

To investigate if person’s current emotional state mediated the effect of rank and unfairness on decisions to work, we used a multi-level mediation analysis approach [32], which nests trial-level observations within upper-level units (individual participants), similarly to the GLME approach described above. The analysis was performed using M3 Mediation Toolbox for MATLAB [33]. Bootstrapping approach, a non-parametric method based on resampling with replacement, was used to estimate the significance of the effects, using the standard 1000 samples [34]. To control for the fact that independent variables in our design were correlated and ensure that the conclusion of the mediation analysis relates specifically to the investigated variable, each mediation model was performed on residuals from a GLME model regressing out the effect of the variable not tested. That is regressing out trial number, and: (i) reward magnitude and rank for the mediation model describing the effect of unfairness, or (ii) reward magnitude and unfairness for the mediation model describing the effect of rank; on both feelings and decisions to work. Prior to the analysis, feelings ratings were transformed to range from 0 to 1, with 0 indicating a low score (i.e., very unhappy).

## Results Experiments 2 and 3

### Opportunity gaps reduce the motivation to work

Across two onsite experiments, participants chose to work on 54% of trials. To test whether the hypothesized factors influenced participants’ choices, we used a generalized linear mixed-effects model (GLME) predicting decisions to work for reward on every trial from unfairness level of all offers, rank of individual’s offered reward (from 1 to 5), and the absolute value of the offered reward (expressed as a power function to account for diminishing marginal utility; see Methods for details). Additionally, we examined if participants reacted to reward offers differently when the minority of individuals are at the top of the distribution and the majority at the bottom or vice versa, by including in the model the signed skewness of the distribution (measured by Adjusted Pearson’s Coefficient of Skewness). The possible effect of fatigue was accounted for by including trial number. All three hypothesized factors significantly influenced decisions to work in exchange for rewards. In particular, the likelihood of pursuing rewards was greater when (i) unfairness was low (β = -0.29, p < 0.001), (ii) rank was high (β = 0.92, p < 0.001) and (iii) absolute reward was high (β = 2.82, p < 0.001). In addition, the likelihood of pursuing rewards decreased over time (β = -1.04, p < 0.001), presumably due to fatigue. Skewness of the distribution did not have a significant effect (β = 0.01, p = 0.92).

To illustrate the impact of unfairness, we calculated each participant’s probability of pursuing rewards at different levels of unfairness and reward magnitudes (based on the estimated fixed and random effects from a GLME model predicting decision to work only from these two factors, setting the other factors to 0). The estimated probabilities were then averaged over participants (Fig 4A). As can be observed, for the same reward magnitude, participants were more likely to work when unfairness was low rather than high. The indifference point (i.e., the reward magnitude for which participants choose to work with 50% probability) was 27.5 points greater for the highest level of unfairness than for the lowest level.

Next, we plotted the likelihood of pursuing rewards for each reward magnitude across the five offer ranks, using the same method as above. As can be observed in Fig 4B the likelihood of pursuing rewards was greater when the rank of the offer is high than when it was low for the same absolute value of the reward. For the lowest rank, participants required an additional 66.4 points to be indifferent on whether to pursue reward than for the highest rank.

To illustrate the effect of unfairness and rank in isolation from the reward magnitude, we plotted the residuals from the above GLME model with the effect of unfairness and rank set to 0. These residuals were then divided into five ranks and two levels of unfairness (high and low based on a median split; Fig 4C). This exercise demonstrates that participants were less likely to work when unfairness was high (red line) than low (blue line) across different ranks. Moreover, participants were more likely to work when the rank of their reward offer was high than when it was low, across different levels of unfairness.

While large unfairness in the group had a negative effect on motivation, it may be that when looking downwards at the less fortunate, large unfairness might increase motivation. To test for this possibility, we added to the above GLME model two covariates for each subject and trial: the sum of distances between the participant and everyone below them (advantageous inequality) and the sum of the distances between the participant and everyone above them (disadvantageous inequality). While all three main effects from the original model remained significant (unfairness: β = -0.33, p < 0.001; rank: β = 0.87, p < 0.001; absolute reward: β = 2.67, p < 0.001), neither upward (β = -0.04, p = 0.67) nor downward (β = -0.29, p = 0.08) comparisons significantly influenced the willingness to work. In other words, while the relative ranking of a participant’s pay offer affects motivation, as does the general level of unfairness, once we account for these two factors, having people’s pay be at a greater distance from others’ in either direction does not additionally impact their willingness to work.

Finally, we compared the original model to two well-known models in the literature that respectively describe the effect of relative value and inequality on utility: (i) the adaptation model, which is based on the assumption that people compare their income to an average value for their reference group [30], and (ii) the Fehr-Schmidt inequality aversion model, which assumes that people have a separate reaction to advantageous and disadvantageous inequality [31]. In both, we include absolute reward and trial number as covariates. Our original model (the ‘rank-unfairness model’) (BIC = 3661.9) outperformed both the adaptation model (BIC = 3765.8) and the Fehr-Schmidt inequality model (BIC = 3780.1), as well as models consisting of only rank (BIC = 3681.3), only unfairness (BIC = 3687.3), or only absolute reward (BIC = 3833.4). Together, the results suggest that high unfairness, low rank and low absolute reward all have significant, negative and independent effects on the willingness to work and that both unfairness and relative value components are necessary to explain the reactions to unequal opportunities.

Across two experiments, we manipulated the level of uncertainty about the monetary value of points by either disclosing or not disclosing the exchange rate (£ per point). To test if the effects differed in these two cases, we added to our GLME model interaction effects between the version of the experiment and the three main factors: rank, unfairness, and absolute reward. We found that the effect of rank and unfairness was stronger when the value of points was unknown than when it was known (interaction with rank: β = 0.97, p < 0.01; interaction with unfairness: β = -0.27, p = 0.019), while remaining significant in both experiments (see Supporting Information). The effect of absolute reward was weaker when the value of points was unknown than when it was known (interaction between experiment version and absolute reward: β = -1.7, p < 0.001). This suggests that participants relied more heavily on social context when they were uncertain about monetary value.

### Feelings partially mediate the effects of opportunity gaps on decisions to exert effort

To examine whether feelings mediated the effects of opportunity gaps on decisions to work, we performed two multi-level mediation analyses. Each of the mediation analysis examined whether feelings mediate the effect of one of the factors identified above (i.e., rank or unfairness) while controlling for the absolute reward magnitude, trial number and the other factor.

**Fig 5:**
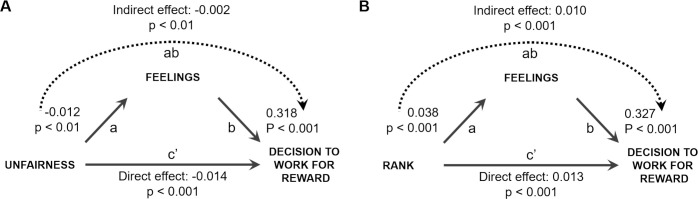
Feelings partially mediate the effect of opportunity gaps on decisions to work for the reward. We examined whether the effect of the two components of the motivational response to opportunity gaps, that is (A) unfairness and (B) rank, were mediated by feelings. In both cases, we controlled for the absolute reward, trial number and either rank (A) or unfairness (B) respectively. In both cases, we found a significant indirect effect and direct effect (which represents the influence of the given factor on decision to work, while controlling for the indirect effect), suggesting that feelings partially mediate the influence of each of the factors on decisions to work.

We found that the effects of unfairness and rank on decision to work were both partially mediated by feelings (see Fig 5). First, as we already reported, low unfairness and high rank were related to greater likelihood to work (total effect: unfairness: β = -0.019, p < 0.001; rank: β = 0.029, p < 0.001). This effect was partially mediated by feelings (path ab: unfairness: β = -0.002, p < 0.001; rank: β = 0.010, p <0.001) with positive feelings related to low unfairness and high rank (path a: unfairness: β = -0.012, p < 0.001; rank: β = 0.038, p <0.001). Additionally, feelings predicted decisions to work even when unfairness and rank were accounted for (path b: unfairness: β = 0.318, p < 0.001; rank: β = 0.327, p <0.001). This suggests that incidental fluctuations of feelings, unrelated to task variables, also had a unique effect on the decision to work. Conversely, the two task related variables had direct effect on the decision to work that could not be accounted for by changes in feelings (path c’: unfairness: β = -0.014, p < 0.01; rank: β = 0.013, p <0.001).

## Discussion

Circumstances beyond a person’s control, such as socio-economic status at birth, often determine the rewards available to a person for their efforts. In the current study, we investigated how decisions to work are altered by a person’s awareness that some people in their group were luckier than others in the rewards they were offered for performing the same task. We hypothesized that the motivation to work would be influenced by the violation of the fairness principle and relative valuation of rewards. Across three experiments, we found that unfair distribution of rewards between group members had a negative impact on the decision to work not only of disadvantaged individuals but also of advantaged individuals. Specifically, high unfairness was related to a reduction in the likelihood that participants agreed to work for their reward irrespective of the magnitude of their reward and their relative position in the distribution. This is despite such refusal reducing the likelihood of receiving a bonus while having no impact on the rewards received by others.

Second, the likelihood of agreeing to work in exchange for reward was reduced when the rank of the offer was low and vice versa (i.e., higher rank was related to greater motivation to work), irrespective of the actual magnitude of the offered reward. The third factor modulating motivation was the absolute reward itself. The fact that absolute reward magnitude exerted influence even when controlling for the level of unfairness and offer rank suggests that while people do care about the rewards of others, they only partially adapt to present social context when deciding whether to work [35].

We find that the rank-unfairness model outperformed the adaptation [30] and inequality-aversion models [31] in explaining participants’ reactions to opportunity-gaps. The adaptation model assumes that people focus on the difference between their reward and the average reward, while the inequality aversion model assumes that people focus on two types of inequality: less heavily weighted advantageous and more heavily weighted disadvantageous inequality. The advantageous inequality is based on the absolute difference between a person’s reward and all other worse rewards, and the disadvantageous inequality is based on the difference between a person’s reward and all other better rewards. All three models predict an increase of motivation with increasing relative value, but the rank-unfairness model is based on ordinal rather than absolute comparisons. Predictions of these model substantially diverge for the effect of statistical dispersion: the rank-unfairness model predicts a uniform decrease of motivation with increased statistical dispersion across all different ranks, while the inequality aversion model predicts a greater drop of motivation for people with lower than higher ranks. On the other hand, the adaption model predicts that person at the top should always be more motivated by an increasing statistical dispersion, as statistical dispersion is associated with greater deviation of their reward from the mean. In both cases, the observed pattern of results is more consistent with the predictions of the rank-unfairness model than the alternatives.

By manipulating the unfairness of offers, offer’s rank and absolute reward in the second and third experiment, we were able to dissociate the influence of each of the three factors within the same individual. By doing so, we overcome a difficulty in studying these variables in the “real-world”, where individuals with different traits or experiences may populate different parts of the distribution [36]—making it difficult to isolate the influence of these components from factors correlating with them, such as negative effects of stereotypes on aspirations [37, 38]. Together, these findings suggest that individuals who are offered less than others are disadvantaged not only because the absolute reward they can possibly obtain is lower, but also because they might suffer from a motivational cost that reduces the likelihood of pursuing the rewards that are within their reach. The latter may be due to a lower relative value of their rewards and a demotivating effect of participating in a situation that seems unfair.

Importantly, because the decisions to work were made in private and did not affect others, the observed effect of unfairness on motivation cannot be attributed to reputation concerns [39], reciprocity [40] or retribution motives [41]. Instead, our results suggest that unfairness and rank exert their effect on motivation partially by influencing experienced feelings. We report a mediation that includes two links: the first is between each of the two factors (unfairness and low rank) and negative feelings; and the second between negative feelings and a reduction in the willingness to exert effort. As for the first link, high unfairness and low rank each triggered negative feelings even when controlling for the magnitude of the reward offered. The negative impact of opportunity gaps on feelings supports the notion that the perception of unfairness is reflected in emotional response [20] and thus carries a cost to one’s psychological well-being. The finding that rank influenced experienced feelings is consistent with studies showing that well-being measures are influenced by a person’s standing relative to others [16–18].

The second link is between feelings and the willingness to work for the reward. Although the idea that unhappiness is related to low motivation is intuitive, there has not been conclusive evidence for it in healthy individuals (for review see: [42]). Past studies have mostly examined the relationship between mood and performance level, rather than the decision to engage in effort altogether, and produced mixed results. While some researchers found a beneficial effect of positive mood induction on performance [43], others found that positive and negative emotions can improve or impair performance depending on the nature of the task [44–47]. With regards to the motivation to pursue rewards, we find that unhappiness has a negative effect. Such an effect could be explained by the negative influence of bad mood on the perceived value of rewards, as suggested by previous experimental studies [21, 48]. Alternatively, rather than playing a causal role, lower happiness in our study could simply index reaction to lower the subjective value of offered rewards [49]. The effects of rank and unfairness were also observed in the first experiment, despite not asking participants about their current emotional state. This suggests that the influence of unfairness and rank on motivation is not conditional on prompted introspection.

The mediatory effect of feelings in a relationship between unfairness and willingness to work for reward was partial, suggesting that additional mechanisms drive the negative influence of unfairness on motivation. One such possibility is that participants use information about the social environment to resolve uncertainty about the value of their offers. In line with this suggestion, we found that in the condition in which the value of points was unknown, the effects of rank and unfairness were stronger than when the value of points was known.

Our study may have implications for people’s decisions and behaviour outside the lab. We speculate that negative feelings caused by arbitrary reward disparities might be one reason why disadvantaged individuals are more likely to suffer from anxiety and depression [50–52]. Furthermore, decreased motivation caused by unfairness and low relative position might make upward mobility particularly difficult, contributing to sustained poverty among disadvantaged groups [9–11]. As such, the motivational phenomenon described in this study constitutes another example of a poverty-trap, that is a situation where having worse prospects triggers additional mechanisms ensuring that a person remains poor. It also suggests that any observed signs of decreased motivation among disadvantaged groups might be situational, rather than stemming from an individual’s characteristics and could be a potential target of interventions.

The instructions of the studies made clear to participants that they had no control over the magnitude of the rewards offered. In contrast, in many everyday situations, there is ambiguity about the role of randomness in success. Previous studies have shown that in such ambiguous situations, those who are advantaged are more likely to assume that their economic position is a result of talent and effort, while those who are disadvantaged assume it is a result of external circumstances [53, 54]. It remains to be tested whether similar effects to those reported here would be observed in such situations.

While past studies have suggested that people are generally averse to unfair distributions of rewards, here we uncover their consequences beyond distribution preferences [31, 55] or impact on the affective state [20, 56]. We show that unequal opportunities have a negative influence on the motivation to work for the reward of not only disadvantaged individuals but also of others around them. Our findings provide an empirical framework for considering the impact of opportunity gaps on individuals, organizations, and societies, suggesting they can trigger psychological dynamics that hurt the productivity of all involved.

## Supporting information

S1 File Supporting methods and data analysis. (DOCX)
