# Supplementary material for: The motivational cost of inequality: Opportunity gaps reduce the willingness to work
Source: PLoS One. 2020 Sep 4;15(9):e0237914. doi: 10.1371/journal.pone.0237914 (PMC7473543; doi:10.1371/journal.pone.0237914)
Supplement: S1 File — (DOCX) [file pone.0237914.s001.docx]

**S1 Table. Distributions of payment offers between participants (expressed in points) for each trial presented in Experiments 2 and 3.**

| Rank | | | | |
| --- | --- | --- | --- | --- |
| 5^th^ | **4^th^** | **3^rd^** | **2^nd^** | **1^st^** |
| 25 | 31 | 36 | 42 | 54 |
| 22 | 29 | 35 | 42 | 59 |
| 19 | 27 | 34 | 43 | 65 |
| 17 | 25 | 33 | 44 | 73 |
| 15 | 23 | 32 | 45 | 82 |
| 13 | 21 | 31 | 47 | 94 |
| 10 | 19 | 30 | 48 | 110 |
| 8 | 17 | 29 | 50 | 129 |
| 7 | 15 | 28 | 52 | 155 |
| 5 | 14 | 27 | 55 | 189 |
| 34 | 44 | 51 | 60 | 79 |
| 30 | 41 | 50 | 62 | 88 |
| 27 | 39 | 49 | 64 | 98 |
| 23 | 36 | 48 | 66 | 111 |
| 20 | 34 | 47 | 68 | 127 |
| 17 | 31 | 46 | 71 | 146 |
| 15 | 29 | 46 | 74 | 170 |
| 12 | 26 | 45 | 77 | 201 |
| 10 | 24 | 44 | 81 | 242 |
| 7 | 21 | 43 | 87 | 297 |
| 48 | 64 | 76 | 90 | 119 |
| 43 | 60 | 75 | 93 | 133 |
| 38 | 57 | 74 | 96 | 150 |
| 33 | 53 | 73 | 100 | 170 |
| 29 | 50 | 72 | 104 | 196 |
| 25 | 47 | 71 | 109 | 226 |
| 21 | 43 | 70 | 114 | 265 |
| 18 | 40 | 69 | 120 | 315 |
| 15 | 37 | 68 | 127 | 379 |
| 12 | 33 | 67 | 136 | 466 |
| 25 | 37 | 43 | 48 | 54 |
| 22 | 39 | 46 | 52 | 59 |
| 19 | 41 | 50 | 57 | 65 |
| 17 | 46 | 57 | 65 | 73 |
| 15 | 52 | 65 | 74 | 82 |
| 13 | 60 | 76 | 86 | 94 |
| 10 | 72 | 90 | 101 | 110 |
| 8 | 87 | 108 | 120 | 129 |
| 7 | 110 | 134 | 147 | 155 |
| 5 | 139 | 167 | 180 | 189 |
| 34 | 53 | 62 | 69 | 79 |
| 30 | 56 | 68 | 77 | 88 |
| 27 | 61 | 76 | 86 | 98 |
| 23 | 68 | 86 | 98 | 111 |
| 20 | 79 | 100 | 113 | 127 |
| 17 | 92 | 117 | 132 | 146 |
| 15 | 111 | 139 | 156 | 170 |
| 12 | 136 | 168 | 187 | 201 |
| 10 | 171 | 208 | 228 | 242 |
| 7 | 217 | 261 | 283 | 297 |
| 48 | 77 | 91 | 103 | 119 |
| 43 | 83 | 101 | 116 | 133 |
| 38 | 92 | 114 | 131 | 150 |
| 33 | 103 | 130 | 150 | 170 |
| 29 | 121 | 153 | 175 | 196 |
| 25 | 142 | 180 | 204 | 226 |
| 21 | 172 | 216 | 243 | 265 |
| 18 | 213 | 264 | 293 | 315 |
| 15 | 267 | 326 | 357 | 379 |
| 12 | 342 | 411 | 445 | 466 |

**S2 Table. Distributions of payment offers between participants (expressed in pence) for each condition in Experiment 1.**

| Offer rank | Fair distribution | | | | | Unfair distribution | | | | |
| --- | --- | --- | --- | --- | --- | --- | --- | --- | --- | --- |
| 1^st^ | 8 | 11 | 14 | 17 | **24** | 1 | 3 | 5 | 8 | **24** |
| 2^nd^ | 12 | 16 | 19 | **24** | 34 | 4 | 8 | 13 | **24** | 73 |
| 3^rd^ | 16 | 21 | **24** | 32 | 45 | 8 | 15 | **24** | 45 | 136 |
| 4^th^ | 18 | **24** | 29 | 36 | 51 | 12 | **24** | 41 | 74 | 225 |
| 5^th^ | **24** | 32 | 39 | 48 | 69 | **24** | 46 | 80 | 144 | 435 |

**S3 Table**. **Influence of unfairness, rank and absolute reward on experienced feelings.** GLME model predicting self-reported feelings.

|  | Coefficient (SE) | T-stat | P-value |
| --- | --- | --- | --- |
| Intercept | 0.45(0.015) | 29.94 | < 0.0001 |
| Trial | -0.052(0.006) | -8.44 | < 0.0001 |
| Absolute reward | 0.095(0.008) | 11.85 | < 0.0001 |
| Rank | 0.070(0.009) | 8.08 | < 0.0001 |
| Unfairness | -0.011(0.004) | -3.60 | < 0.0001 |

**S4 Table**. **GLME model predicting decisions to pursue rewards in Experiment 2 (value of points known).**

|  | Coefficient (SE) | T-stat | P-value |
| --- | --- | --- | --- |
| Intercept | 1.10(0.60) | 1.83 | < 0.01 |
| Trial | -1.45(0.17) | -8.37 | < 0.0001 |
| Absolute reward | 4.33(0.42) | 10.27 | < 0.0001 |
| Rank | 0.37(0.12) | 3.13 | < 0.0001 |
| Unfairness | -0.14(0.09) | -1.98 | 0.058 |

**S5 Table**. **GLME model of decisions to pursue rewards, Experiment 3 (value of points unknown).**

|  | Coefficient (SE) | T-stat | P-value |
| --- | --- | --- | --- |
| Intercept | 0.45 (0.35) | 1.29 | 0.20 |
| Trial | -0.73(0.13) | -9.77 | < 0.0001 |
| Absolute reward | 1.70(0.22) | 7.96 | < 0.0001 |
| Rank | 1.33(0.14) | 9.40 | < 0.0001 |
| Unfairness | -0.41 (0.09) | -4.40 | < 0.0001 |
